# Quality of Life One Year after Coronary Artery Bypass Graft Surgery

**Published:** 2011-03-01

**Authors:** H R Taghipour, M H Naseri, R Safiarian, Y Dadjoo, B Pishgoo, H A Mohebbi, L Daftari Besheli, M Malekzadeh, A Kabir

**Affiliations:** 1 Trauma Research Center, Department of Cardiology and Cardiothoracic Surgery, Bagiyatallah University of Medical Sciences, Tehran, Iran; 2 Department of Epidemiology, Shahid Beheshti University of Medical Sciences, Tehran, Iran; 3 Center for Educational Research in Medical Sciences, Tehran University of Medical Sciences, Tehran, Iran

**Keywords:** Quality of Life, Coronary artery bypass, Questionnaires, Iran

## Abstract

**Background:**

Coronary artery bypass graft (CABG) is a treatment strategy to relieve the symptoms of coronary artery disease (CAD). Based on determining the long term outcome of CABG using SF-36 Health Related Quality Of Life (HRQOL) questionnaire, the present study was conducted in our center to determine the CABG results one-year after the operation.

**Methods:**

Between March 2005 and August 2009, 112 patients with coronary heart disease (CHD) who underwent coronary artery bypass graft (CABG) were enrolled. Patients completed SF-36 HRQOL general health status questionnaire. Stepwise multiple linear regression models were used to detect independent variables predicting changes in each eight subscales of SF-36 questionnaire.

**Results:**

The mean age of patients was 61.4±0.9 years and most of them were male with three vessel diseases that were on pump CABG. The mean physical and mental component summary scores were 59.5±0.9 and 60.2±0.9, respectively. Physical functioning (PF) and role physical (RP) improved in males. Regression models showed that there were some statistical models with low R-square to predict role emotional (RE), general health (GH), PF and RP according to ejection fraction after surgery, diabetes, pump type of CABG and male gender.

**Conclusion:**

CABG has led to higher and more satisfactory outcomes for PF, RP and RE but lower in other scales comparing with normative data of the society and one-year post-operative scores of other studies. It could mostly be attributed to unmodified risk factors and progression of existing comorbidities.

## Introduction

Cardiovascular diseases (CVD) are still one of the leading causes of morbidity and mortality worldwide. In Iran, the most common form of this disease is coronary artery disease (CAD) with a prevalence of 37.5% in women and 22.2% in men based on the Rose questionnaire and Minnesota coding [1] and accounts for nearly 50% of all deaths per year.[2] Coronary Artery Bypass Graft (CABG) surgery is an accepted treatment procedure to improve the symptoms of CAD. The number of patients undergoing this procedure has an increasing trend in most countries. [3] However, one study in Iran showed a 4% decline in the country. [4] Recently, interest in measuring the quality has spread from industries into the health care system too. Traditional evaluation of CABG outcomes has focused on objective measures such as mortality, morbidity and clinical function. [5] But in recent years, the emphasis has directed to include more subjective parameters, especially Health Related Quality Of Life (HRQOL) measures which have obtained increasing popularity as outcome variables in cardiac research. [6] Main domains in HRQOL assessment are physical functioning, emotional status, cognitive performance, social functioning, general perceptions of health and well-being, and disease-specific symptoms. [7] Due to complexity and impossibility of administering lengthy and detailed means for patients with serious medical conditions, the best way to monitor patient’s HRQOL variables is to use a quickly available, validated survey instrument that is easy to complete, process and score, and sensitive enough to detect changes in pain, function, and overall health status at follow up.

The short form, 36 questions (SF-36) general health status survey, is a single 36 item scale recommended by the American Association of Cardiovascular and Pulmonary Rehabilitation (AACVPR) used for evaluating HRQOL in patients with cardiovascular disorders as well as normal population and individuals with various disease states that allows calculation of numerical scores for eight dimensions of health. [8] The SF-36 provides HRQOL information in subscales of physical functioning (PF), role physical (RP), bodily pain (BP), general health (GH), vitality (VT), social functioning (SF), role emotional (RE) and mental health (MH). It is well known that CABG can affect all these aspects. Health-related quality of life before and after CABG surgery has been studied extensively using a variety of generic and disease specific HRQOL instruments. With the use of SF-36, many international surveys have confirmed that HRQOL generally improves after cardiac surgery. [9] Most studies are restricted to short-term follow-ups, although some have followed up patients for longer durations. [10] Yet, one year post CABG surgery, HRQOL improvement is not well confirmed and only a limited number of studies have specifically evaluated patients at one-year follow up using SF-36. [11][12][13]

Regarding this lack of research for measuring longer term outcomes with the use of SF-36 in Iran, the present study was conducted to determine if CABG results into satisfactory outcomes one-year after the operation.

## Materials and Methods 

Between March 2005 and August 2009, 112 patients with coronary heart disease (CHD) who had undergone a coronary artery bypass graft (CABG) were randomly (using EPI software for a simple randomization) selected. Cases were referral of Department of Cardiac Surgery, Baqiyatallah University of Medical Sciences, Tehran, Iran. Considering α=0.05, score of the QOL equal to 70%, precision about 8.5%, and by using estimation of a ratio formula, the sample size was estimated 112 cases.

The inclusion criteria were undergoing CABG at least 12 months ago and signing a written informed consent form to complete the SF-36 questionnaire. The Ethical Committee of Baqiyatallah University of Medical Sciences approved the study protocol. Patients completed SF-36 questionnaire themselves (self administered). In case of illiteracy, one trained secretary helped them to fill the questionnaire. In addition, we assessed demographic variables of age, gender, height, weight, duration of hospitalization, date of enrollment, CHD risk factors (smoking, family history, hypercholesterolemia, hypertension, diabetes), number of diseased and grafted vessels, type of surgery (on pump or off pump), duration of aortic cross clamp and CABG, and ejection fraction before and after surgery according to the medical history, physical examination, laboratory tests and patients’ file if necessary. In addition, to determine left ventricular ejection fraction (LVEF), a transthorasic echocardiography was done by the cardiologists. Normal range of lipids is considered as triglyceride (TG) < 150, low density cholesterol (LDL) < 100, and total cholesterol (TC) < 200 mg/dl for both men and women.

Quality Metric's SF-36v2® Health Survey asks 36 questions to measure functional health and well-being from the patient's point of view. It is a practical, reliable, and valid measure of physical and mental health that can be completed in five to ten minutes. The SF-36v2 provides scores for each of the eight health domains, and psychometrically-based physical component summary (PCS) and mental component summary (MCS) scores, [8] (Table 1). It has previously been validated in Iran. According to Cronbach’s alpha, its reliability has been estimated equal to 0.73 for PCS and 0 .72 for MCS.[14] We followed all patients regularly after surgery. They filled SF-36 questionnaire 12 months or more after CABG.

**Table 1:** Scales, number of items and scale formats for SF-36.

**SF-36**	**Scale (No. of items) a**	**Scale format (No. of items) b**	**Scale score range c**
Physical Component Summary Score (PCS) d	SF-36 Physical Functioning (10) e	3-point scale (10)	0–100
	SF-36 Role – Physical (4)	2-point scale (4)	0–100
	SF-36 Bodily Pain (2)	5-point scale (1), 6-point scale (1) f	0–100
	SF-36 General Health (5)	5-point scale (5)	0–100
Mental Component Summary Score (MCS) g	SF-36 Vitality (4)	6-point scale (4)	0–100
	SF-36 Social Functioning (2) g	5-point scale (2)	0–100
	SF-36 Role – Emotional (3)	2-point scale (3)	0–100
	SF-36 Mental Health (5) g	6-point scale (5)	0–100

^a^ Scales measuring the frequency of CHD symptoms

^b^ The items in the Adverse Effects scale of the CROQ-CABG and CROQ-PTCA differ as the procedures are different

^c^ For each instrument higher scores indicate higher functional levels

^d^ The Physical and Mental Component Summary Scores are scored with weights, and are expressed as t-scores with mean=50 and standard deviation=10

^e^ Scales measuring physical functioning

^f^ Items are recalibrated to the same scale format before summing to calculate the scale score

^g^ Scales measuring the impact of health on psychological and/or social functioning

We used mean±SE for description of quantitative variables. Pearson and Fisher Exact, Chi-Square, student and paired t tests, one-way analysis of variance, Pearson correlation were used for statistical analysis. We used stepwise multiple linear regression models with Pearson correlation coefficient (r) to detect independent variables that can predict changes in each eight components of SF-36 HRQOL questionnaire. Differences and correlations with p<0.05 were considered statistically significant. We used SPSS 13 software (SPSS Inc., Chicago, Illinois, USA) in the analysis.

## Results

The mean age of patients was 61.4±0.9 years and most of them were male. Most of them had three vessel diseases and underwent on pump CABG (Table 2).

**Table 2:** Clinical and sociodemographic characteristics of the patients at baseline.

**Variable **		
Age (Year), mean±SE		61.4±0.9
Male gender, No. (%)		72 (61.5)
Hospitalization (Day), mean±SE		8.2±0.2
Hypercholesterolemia, No. (%)		55 (47)
Smoking, No. (%)		36 (30.8)
Family history, No. (%)		64 (54.7)
Hypertension, No. (%)		59 (50.4)
Diabetes, No. (%)		48 (41)
Number of grafts, No. (%)	1	7 (6)
	2	24 (20.5)
	3	26 (22.2)
	4	54 (46.2)
	5	6 (5.1)
On pump, No. (%)		94 (83.9)
Coronary disease, No. (%)	Single Vessel	7 (6)
	Two Vessel	21 (17.9)
	Three Vessel	89 (76.1)
Aortic cross clamp (minute), mean±SE		39.9±1.5
Cumulative bypass time (minute), mean±SE		67.8±2.2

Ejection fraction (EF) before and after surgery were 44.7±0 .7 and 49±0.6, respectively. Statistical analysis showed that it has increased significantly (p<0.001). The mean physical and mental component summary score of their HRQOL were 59.5±0.9 and 60.2±0.9, respectively. Mean score of eight subscales of HRQOL of patients after one year of CABG was 86.9, 83.3, 74.6, 53.4, 52, 48.3, 47, and 33 for RE, RP, PF, VT, MH, SF, GH, and BP, respectively (Table 3).

**Table 3:** Comparison our results with similar studies.

**Scale**	**Mean score in present study (SE)**	**Mean scores in similar studies (SE)**				
		**UK/2000[11] No.=183**	**Australia/2000 [15] No.=108**	**USA/2002 [12] No.=81**	**Poland/2005 [13] No.=104**	**Iranian Norms (age >45) [14] No.=1129**
SF-36 Physical Functioning (PF)	74.6 (2.6)	60 (31-85) a	76.5 (2.1)	75.5 (2.6)	63.5 (2.7)	70.7 (0.7)
SF-36 Role – Physical (RP)	83.3 (3)	25 (0-100) a	60.6 (4.1)	53.7 (5)	60.1 (4)	51.5 (1.2)
SF-36 Bodily Pain (BP)	33 (2.3)	61 (2.1)	72.1 (2.5)	72.8 (2.3)	75 (2.2)	67.9 (0.8)
SF-36 General Health (GH)	47 (0.9)	55 (1.8)	69.6 (2.2)	63.3 (2.5)	43.6 (1.7)	56.5 (0.6)
SF-36 Vitality (VT)(Energy/fatigue)	53.4 (0.9)	48 (1.8)	61.7 (2.1)	60.4 (2.4)	57.3 (2.4)	59.6 (0.5)
SF-36 Social Functioning (SF)	48.3 (1.2)	67 (44-100) a	79.8 (2.5)	84 (2.4)	76.2 (2.4)	66.7 (0.8)
SF-36 Role – Emotional (RE)	86.9 (2.8)	50 (0-100) a	70.7 (3.8)	71.6 (4.4)	64.7 (3.8)	56.6 (1.3)
SF-36 Mental Health (MH)	52 (0.8)	66 (1.3)	75.3 (1.9)	79.3 (1.7)	63.3 (2.1)	63.4 (0.5)

^a^ Medians with the interquartile range in parentheses

The male to female ratio was 2.6 to one. PF (81.5±2.9 vs 63.8±4.4, p=0.001) and RP (89±3.2 vs 74.4±5.6, p=0.027) were better in males. Mean physical component summary score was significantly higher in males (62±1 vs 55.5±1.6, p=0.001) but mean mental component summary score had no difference in both genders. Type of surgery had a significant effect on general health. Patients on pump surgery had a better general health status than off pump subjects (47.9±0.9 vs 42.5±2.2, p=0.024).

Patients with DM had higher score of emotional role in comparison with cases without DM (94.7±2.6 vs 81.9±4.3, p=0.012). EF after CABG was negatively correlated with emotional role (r=-0.242, p=0.01). Physical functioning was also negatively correlated with EF before (r=-0.220, p=0.021) and EF after (r=-0.213, p=0.025) CABG. The regression models (Table 4) showed that there are some statistical models to predict RE, GH, PF and RP according to EF after surgery, diabetes mellitus, on pump type of CABG and male gender. Since, r-square of all models are low, these are considered as weak models. According to first model, presence of DM and lower EF after surgery predict a higher emotional role (p<0.001, R-square=0.2). The second model showed that on pump type of CABG is an independent variable to predict higher general health status (p=0.002, R-square=0.1) of the patients. The two other models showed that male gender would independently predict higher PF (p<0.001, R-square=0.12) and RP (p=0.017, R-square=0.05).

**Table 4:** Predictors of some part of HRQOL of the patients according to the linear regression analysis.

** Dependent variable **	** Independent variable **			** P value of the model **	** R-square **
	** Variable **	** Standardized Beta **	** Sig **		
RE	EF after surgery	-0.387	<0.001	<0.001	0.19
	Diabetes	0.234	0.01		
GH	On pump CABG	0.286	0.002	0.002	0.08
PF	Male Gender	0.348	<0.001	<0.001	0.12
RP	Male gender	0.226	0.017	0.017	0.05

## Discussion

In this study, one year post CABG, SF36 results showed that subscales of BP, GH, VT, SF and MH were lower than Iranian’s normative data; but, subscales of SF, RP and RE were higher. Additionally, in comparison with other similar studies, RP and RE scales were higher and other subscales were mostly lower (Table 3). Furthermore, men had higher scores in PF and PR than women in this study and patients with on pump surgery and those with diabetes mellitus had a higher GH and RE respectively.

Comparison of our results with one-year post CABG findings of four different studies conducted in UK, Australia, USA and Poland separately,[11][12][13][15] revealed that the items of PF, RP and RE were equal or significantly higher in our study; however, BP, SF, VT, GH and MH were lower. As these studies were from various parts of the world including Europe, North America and Australia, this study can be considered as the representative of Middle East. To explain these differences in HRQOL between different studies, it should be considered that the norms of HRQOL are different in these regions and the Iranian norms in most scales are lower than the ones in most of the European, American and Australian countries [12][14][15][16] which enforce on higher HRQOL scores in Iranian patients undergoing CABG in comparison with these countries. To the best of our knowledge, there was no similar study in the other Middle East countries for comparison.

Other reasons for the differences between our study and others can be due to the fact that, the HRQOL would not improve in a linear way for all patients following CABG. It is also due to higher rates of comorbidities and three vessel patients in our study. As there were no pre-operative baseline data for SF-36, the results of this study were compared with Iranian’s normative data which was published by Montazeri et al., [14] in 2005. They assessed HRQOL in random samples of 4163 healthy individual aged 15 years and over with Iranian version of SF-36. Because the age range of our study population was between 44 and 84 years, we compared our results with the age groups of ≥45 years old of the mentioned study. The scales of RP, RE and PF in our study were higher than Iran’s normative data. As the RP and RE reflect the problems encountering with daily activities and works which can be affected by physical health and emotional problems, the higher score means denote to a better situation in these activities. It seems that patients’ self esteem would be rebuilt after operation; they can undertake their activities and overcome their job problems.

About the PF scale, although it was higher in our study, the difference was not significant and its range was almost the same in the two studies. Finally for other scales, our results were lower than norms and the difference was more significant in BP scale. Comparison of these one-year postoperative scores with Iranian healthy individual’s normative data indicated that our patients had lower measures in most health domains except in PF, RP and RE.

Subjects in our study had a worse mental health condition compared with their physical health and the mental health reported in other studies. One possible explanation of these differences is perhaps the continuation of existing comorbidities in our population which could negatively influence the subjective and multifactorial nature of the concept of HRQOL. It means that the patients probably have not properly modified their risk factors after the operation. Other reasons could be the poor participation in cardiac rehabilitation programs which have proved to have a great effect on quality of life after the operation [17] or noncompliance with prescribed medications and occupational advices and finally it could be as a result of disease burden of coronary artery disease which even after surgery will not increase more than normative scores. These results are to some extent similar to other studies that have compared their findings with population norms using the SF-36. They found that longer-term HRQOL after CABG is commonly worse than that of the general population in most scales. [9][18]

In our study, men had higher scores in the subscales of PF and RE. With difference in significant scales, results of this study are in line with other surveys regarding higher scores in men.[19][20] It can be contributed to lower preoperative scores in women than men, more limited physical and psychological gains in women than in men after CABG and achieving less complete revascularization [20] and higher graft occlusion rate [21] in women than men. Moreover, our results showed that patients of on pump surgery had significantly higher GH than off pump ones. In studies comparing the quality of life of on pump versus off pump in CABG patients, [22][23] there were no significant differences between two groups of patients in all domains of SF-36, except one study [23] which reported slightly better outcomes in the domain of RP in on pump group than the off pump group five years postoperation. It sounds logical that natural procedures like on pump had better QOL; however, future projects should work more on this issue.

In our study, the patients with diabetes mellitus had higher RE in comparison with non diabetic patients while according to another study, [11] the diabetic patients had worse quality of life in most domains of SF-36 in comparison with non diabetic subjects. In our study, EF after CABG was negatively correlated with RE and there was an inverse relationship between PF and EF before and after CABG as well. One study showed that the lower preoperative EF had a negative effect on quality of life after CABG [24] and in another study, improvement in symptoms and QOL were not dependent on preoperative EF. [25]

The authors of this study recommend a regular and strict follow up for participation of patients in cardiac rehabilitation programs which have been proved to have a great effect on quality of life of patients after the surgery, [17] the point which has not been put into practice in this study. Furthermore, risk factor modification and following up of patients to use the prescribed drugs regularly are of great importance and have a great effect on the patients’ HRQOL. We were able to answer the questions on different aspects of the QOL of patients, what would happen after surgery and details that affect on all aspects of patients’ lives. Moreover, we could also predict some parameters of HRQOL according to some easy to understand or measurable variables such as diabetes mellitus, gender, and type (on pump/off pump) of surgery before CABG.

One of the limitations of this study was that no pre-operative baseline SF-36 data was available. Therefore, a comparison of pre-operative and post-operative data was not possible, so it was difficult to draw conclusions for post CABG while no baseline were available.The other problem was the weakness of the SF-36 which could not control the effects of comorbidities which may have resulted in various studies. HRQOL of the patients after one year from CABG was relatively good. In this survey, CABG has led to higher and more satisfactory outcomes for PF, RP and RE but lower in other scales comparing with normative data of the society and one-year post-operative scores of other studies. It could mostly be attributed to unmodified risk factors and progression of existing comorbidities.
